# Practice Patterns on the Incorporation of Integrative Medicine Into the Oncologic Care of Patients With Cancer

**DOI:** 10.1177/15347354231213045

**Published:** 2023-11-18

**Authors:** Santhosshi Narayanan, Wenli Liu, Gabriel Lopez, Bryan Fellman, Akhila Reddy, Ishwaria M. Subbiah, Lorenzo Cohen, Eduardo Bruera

**Affiliations:** 1 The University of Texas MD Anderson Cancer Center, Houston, TX, USA

**Keywords:** integrative oncology, complementary and alternative treatments, cancer, integrative medicine, patient reported outcomes, implementation research

## Abstract

**Background:**

With rising interest in complementary approaches to symptom management, awareness of real-world practice patterns in the incorporation of integrative oncology (IO) into cancer care is limited. Therefore, we examined the reasons for referral, symptom burdens, and clinical outcomes for cancer patients who underwent initial IO consultations.

**Methods:**

The records of adult patients with cancer who underwent initial outpatient IO consultations at our cancer center for a representative 10-day period at the start of each month for 12 months starting January 1, 2017, were reviewed retrospectively. Patient demographic and medical characteristics and outpatient IO consultation details, including patient-reported outcome measures of symptom burden, were extracted. Descriptive summary statistics and logistic regression were used to analyze the data.

**Results:**

Among the 473 study patients, 71% were women, breast cancer (42%) was the most common cancer type, and 31% had metastatic cancer. Referring clinicians listed an integrative approach (57%) as the most common reason for referral, followed by diet (26%), pain (19%), discussion of herbs and supplements (18%), and stress (18%). In comparison, patients listed their primary concerns as diet (16%), pain (15%), and an integrative approach to overall health (11%). After the IO consultations, the highest likelihood of subsequent recommendations were acupuncture for hot flashes (odds ratio [OR], *P* = .002) or peripheral neuropathy (OR = 6.59, *P* < .001), oncology massage for pain (OR = 3.04, *P* < .001), psychology referral for patient’s self-reported anxiety (OR = 2.35, *P* < .001), and mind-body therapies for stress (OR = 2.57, *P* < .001).

**Conclusion:**

Cancer patients’ top concerns regarding IO consultation may not always match providers’ reasons for referral. Longitudinal data analysis is needed to determine the effect of integrative interventions on symptom burden.

## Introduction

Integrative oncology (IO) is a field that has grown rapidly over the past decade, and most leading U.S. cancer centers are providing complementary and integrative therapies to their patients.^1^ Witt et al^2^ defined IO as “a patient-centered, evidence-informed approach to health care that utilizes mind-body therapies, natural products, and lifestyle modifications from different traditions alongside conventional cancer treatments.” They added that IO “aims to optimize health, quality of life, and clinical outcomes across the cancer care continuum and to empower people to prevent cancer and become active participants before, during, and beyond cancer treatment.” Recommendations for IO and integrative therapies are now included in American Society of Clinical Oncology (ASCO) and National Comprehensive Cancer Network guidelines.^3-6^ This illustrates the increasing interest in studies of real-world practice patterns and IO recommendations.

According to the results of the American Society of Clinical Oncology’s second National Cancer Opinion Survey in 2018, 4 in 10 Americans believe that “alternative treatments” may cure cancer and that such treatments often include herbs and supplements.^7^ Unfortunately, in a survey of 392 oncologists (American Society of Clinical Oncology members), two-thirds reported not having enough knowledge to discuss the area of herbs and supplements with their patients.^8^ This lack of knowledge can lead patients to make independent decisions regarding natural products and alternative therapies while rejecting conventional cancer treatments. Acknowledgment that such choices, which are based on unsubstantiated claims, may not align with the best interests of the patients is crucial. For example, a patient may want to discuss recommendations regarding herbs and supplements to help manage symptoms such as arthralgia caused by treatment with aromatase inhibitors, but the evidence is often stronger for other therapeutic modalities, such as acupuncture.^9^ An IO consultation is an opportunity to identify and evaluate symptoms that can be treated with appropriate integrative approaches. Furthermore, in our clinical practice, we educate patients on the appropriate integrative therapies and realign their expectations to help them make informed decisions about their care.^10,11^

Currently, a clear understanding of the real-world interplay of the differences in patient expectations, physician referral reasons, and clinical recommendations arising from IO consultations is lacking. Therefore, we explored the reasons for referral to IO by clinicians, patient concerns for seeking IO consultations, recommendations in initial IO consultations, and factors associated with recommendation of common integrative therapies.

## Methods

### Patients and Outcomes

A retrospective study of cancer patients seen for initial consultation in the ambulatory IO clinic at our large comprehensive cancer center was conducted. Encounters analyzed were limited to those taking place during the first 10 days of each month from January 1, 2017, through January 1, 2018. Including the first 10 days of each month ensured that our sample was representative of all patients seen at the IO clinic allowing us to obtain a statistically robust data set for accurate outcome measurements.

All patients 18 years or older with a cancer diagnosis were included in the study. The final sample consisted of 473 patients. Waivers of informed consent were obtained, and the study was approved by The University of Texas MD Anderson Cancer Center Institutional Review Board (PA 18-0892). Data were collected via review of each patient’s electronic medical record.

Our primary outcome was identification of the clinicians’ referral reasons for IO consultation and the patients’ self-reported concerns at the time of the IO encounters as described in the medical record. On the electronic heath record consultation order referral form, referring providers were given the following options as reasons for referral to an IO consultation: diet, exercise, weight management, stress, relaxation, anxiety, depression, nausea, appetite, fatigue, hot flashes, pain, an integrative approach, overall health, sleep, dry mouth, herbs and supplements, and neuropathy. More than one reason could be selected by the referring clinician. When patients arrived for their IO consultations and at follow-up, they listed their self-reported concerns using a modified Measure Yourself Concerns and Wellbeing (MYCaW) assessment.^12^ Patients reported their top 2 concerns regarding their IO encounters from a list of available topic areas, including an integrative/holistic approach, herb/supplements, diet/nutrition, pain, overall health, and stress/anxiety as well as an “other” category.

Our secondary outcome was evaluation of the factors associated with recommendation of IO clinical services after consultations. Before each IO consultation and at follow-up, patients completed a series of measures to assess symptom burden (Modified Edmonton Symptom Assessment Scale [mESAS]) and quality of life (Patient-Reported Outcomes Measurement Information System-10 [PROMIS-10]), with responses immediately available for review by the health care provider (IO physician and/or nurse practitioner).

The mESAS^13^ consists of 16 items: 10 core symptoms (pain, fatigue, nausea, depression, anxiety, drowsiness, loss of appetite, decreased sense of well-being, shortness of breath, and sleep difficulty) plus an additional 6 items including spiritual distress, financial distress, numbness/tingling, hot flashes, dry mouth, and memory problems. Respondents rate the symptoms they have experienced over the past 24 hours on a scale of 0 to 10, with 10 being the worst. A difference of 1 for an individual item is considered a clinically significant difference in that symptom.^14^ Subscale scoring is performed as follows: the *global distress score* (range, 0-90) is the sum of the pain, fatigue, nausea, drowsiness, appetite, shortness of breath, anxiety, depression, and well-being scores; the *physical distress score* (range, 0-60) is the sum of the pain, fatigue, nausea, drowsiness, appetite, and shortness of breath scores; and the *psychological distress score* (range, 0-20) is the sum of the anxiety and depression scores.

PROMIS-10^15^ is an assessment of global health with 10 self-reported items that are divided into mental health and physical health subscales. Responses are converted into *T*-score values, with *T*-score distributions standardized to the mean for the U.S. population. Higher scores represent better global, mental, or physical health.

During IO consultations at our institution, patients are evaluated comprehensively for physical, mind-body, and social needs in the context of their cancer and medical conditions and referred to IO services. Our IO clinical service providers include board-certified physicians, advanced practice providers such as physician assistants and nurse practitioners, licensed acupuncture therapists, licensed massage therapists, psychologists with doctorate qualifications, a registered dietician, a physical therapist, a certified yoga therapist, and a music therapist. At our IO clinic, personalized care for each patient is prioritized, ensuring that their unique needs are met. To achieve this, individualized integrative care plans that consider various factors are developed. These plans may incorporate acupuncture or massage therapy to address symptom control, health psychology, yoga, meditation, or music therapy to address psychological distress; counseling for healthy lifestyle behaviors like diet and exercise; and referrals to nutritionists, physical therapists, or health psychologists. Additionally, providers engage in discussions regarding the risks of and evidence regarding use of herbs and supplements and alternative treatments pursued or considered by patients. The importance of promoting evidence-based information and fostering open communication between patients and health care professionals is emphasized. Empowering patients with accurate knowledge can help them make informed decisions, ensuring that their chosen paths align with their overall well-being. Our recommendations are consistently tailored to the specific requirements of each patient, considering established guidelines and the latest available evidence.

### Statistical Analyses

Descriptive statistics were used to summarize the patients’ demographic and clinical characteristics. The reasons for referral and patient concerns recorded via the MYCaW instrument were summarized using frequencies and percentages. The mESAS and PROMIS-10 scores were summarized with means, SDs, and ranges. All statistical analyses were performed using Stata/MP software (version 16.0; StataCorp). Logistic regression was used to calculate odds ratios (ORs) for the secondary outcomes. An ESAS score of 4 was used as a cutoff for calculating the OR for symptoms predicting recommendation of integrative interventions based on patients’ self-reported symptom burdens. The required minimum sample size was estimated to be 300 patients to estimate the proportion of reasons for referral with a 95% CI having a half-width of less than or equal to 0.06. Unless otherwise stated, all tests were 2-sided, and *P* levels of <.05 were considered significant.

## Results

Our study population consisted of 473 patients. Table 1 presents their demographic and clinical characteristics. Seventy-one percent of the patients were women; 42% had breast cancer, the most common diagnosis; and 31% had metastatic disease. Patients seen in our IO clinic lived a median distance of 98.0 miles away (interquartile range 15.9-549.9 miles). The mESAS symptom burden and PROMIS-10 data are also shown in Table 1. The highest mean (±SD) baseline mESAS scores (ie, ≥4, indicating worse symptoms) were for sleep (4.9 ± 2.7), fatigue (4.5 ± 2.8), and well-being (4.3 ± 2.6).

**Table 1.** Demographic and Clinical Characteristics of New Patients Seen in an Ambulatory Integrative Oncology Consultation.

Characteristic	No. (N = 473)	%
Age at consult, y		
Mean (SD)	56 (13)	
Median (range)	57 (20-87)	
Distance from MD Anderson, miles		
Mean (SD)	362 (612)	
Median (range)	98 (0-6541)	
Sex		
Female	335	71
Male	138	29
Primary race		
Asian	27	6
Black or African American	54	11
Other/unknown	48	10
White or Caucasian	344	73
Ethnicity		
Hispanic or Latino	58	12
Not Hispanic or Latino	404	85
Unknown/declined to answer	11	2
Cancer diagnosis		
Breast	198	42
CNS	15	3
Endocrine	13	3
Gastrointestinal	53	11
Genitourinary	50	11
Gynecological	20	4
Head and neck	34	7
Hematological	41	9
Lung	27	6
Sarcoma	7	1
Others	15	3
Cancer stage		
Metastatic disease^a^	146	31
ESAS score, mean (SD)		
Anxiety	3.3 (2.9)	
Appetite	3.3 (2.8)	
Depression	2.2 (2.6)	
Drowsiness	2.8 (2.8)	
Dry mouth	2.4 (2.9)	
Fatigue	4.5 (2.8)	
Financial distress	3.0 (3.0)	
Hot flashes	2.2 (3.0)	
Memory	3.7 (2.5)	
Nausea	1.4 (2.3)	
Numbness and tingling	2.8 (3.1)	
Pain	3.5 (3.0)	
Shortness of breath	1.6 (2.4)	
Sleep	4.9 (2.7)	
Spiritual pain	1.4 (2.2)	
Well-being	4.3 (2.6)	
PROMIS10 score,^b^ mean (SD)		
Mental health subscale	12.6 (3.3)	
Physical health subscale	13.1 (3.2)	

Abbreviation: ESAS, Edmonton Symptom Assessment System.

a Not all patients had data for all variables as some patients were still undergoing work-up at the time of consultation.

b PROMIS10 includes a mental health subscale (4-20), physical health scale (4-20), and global health total score (11-50). Higher scores represent better mental, physical, or global health. Responses were converted into *T*-score values; *T*-score distributions were standardized to the US population mean.

**Figure 1.**
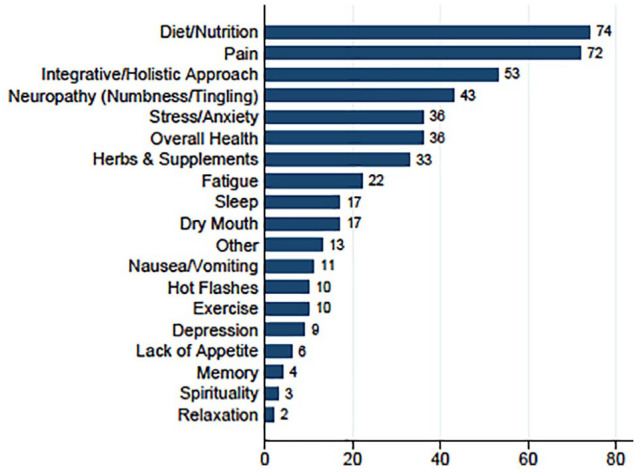
Patients’ primary concern for seeking an integrative oncology consultation (MYCaW1) (%). N = 471 (2 patients did not respond to the question). Abbreviation: MYCaW, Measure Yourself Concerns and Wellbeing.

Table 2 lists the reasons for referral to IO consultation by referring clinicians (physicians or advanced practice providers) in clinical centers throughout the comprehensive cancer center. The most common referral reasons were an integrative approach (57%), diet (26%), pain (19%), herbs and supplements (18%), stress (18%), and overall health (15%). Table 2 also illustrates how a patient’s self-reported concerns at the time of a clinical encounter may not always align with their health care provider’s referral reason. For example, among the 86 patients referred for discussion of herbs and supplements, only 24 mentioned discussing herbs and supplements as one of their top 2 concerns, whereas among the 88 patients referred for pain, a majority (n = 69) reported pain as one of their top 2 concerns. Figure 1 shows the primary self-reported concerns patients wanted addressed during the IO consultations. Diet/nutrition (16%), pain (15%), and an integrative approach (11%) were the most frequently cited concerns.

**Table 2.** Reasons for Provider Referral to Ambulatory Integrative Oncology Consultation and Percentage of Patients whose Concerns Aligned to the Referral Reason.

Reason for referral	Patients referred N (%) (N = 473)^a^	Patients with concern matching referral N (%)^b^
Integrative approach	270 (57)	69 (26)
Diet	124 (26)	55 (44)
Pain	88 (19)	69 (78)
Herbs and supplements	86 (18)	24 (28)
Stress	86 (18)	50 (58)
Overall health	72 (15)	38 (53)
Sleep	50 (11)	29 (57)
Neuropathy	50 (11)	41 (82)
Fatigue	54 (11)	32 (59)
Exercise	46 (10)	20 (43)
Relaxation	30 (9)	10 (33)
Dry mouth	24 (5)	16 (67)
Weight management	22 (5)	0 (0)
Nausea	20 (4)	12 (60)
Hot flashes	21 (4)	12 (57)
Depression	19 (4)	15 (79)
Appetite	13 (3)	7 (54)

a The sum of n is greater than the number of patients as there was often more than one reason for referral for each patient.

b The percentage given is the number of patients citing the concern among all patients referred for that particular concern from the previous column.

Table 3 provides insight into the factors (initial IO referral reason and patient symptom burden reflected by scores of ≥ 4 on ESAS individual items) that predicted recommendations of integrative interventions after IO consultations, such as acupuncture, massage, psychological, and mind-body therapies. Initial referral to IO for management of hot flashes had the highest OR (23.14; *P* = .002) for recommending acupuncture, followed by referral for neuropathy (OR, 6.59; *P* < .001). Self-reported ESAS scores of 4 or higher for fatigue, nausea, neuropathy, hot flashes, pain, sleep, and dry mouth significantly predicted recommendation of acupuncture. Initial IO referrals for stress, relaxation, and pain or ESAS scores of at least 4 for pain and anxiety were significantly associated with recommendation of oncology massage. Referral for stress and ESAS scores of at least 4 for anxiety, depression, fatigue, and nausea were markedly associated with psychology referral. Referral for stress and pain and higher ESAS scores for depression, anxiety, and pain significantly predicted recommendation of mind-body therapies.

**Table 3.** Symptoms Predicting the Recommendations of Integrative Interventions Based on Referral Reason and Patient’s Self-Reported Symptom Burden.

Integrative interventions	Initial IO referral reason	*P* value	Symptom scores (ESAS ≥ 4)	*P* value
	Odds ratio (95% CI)		Odds ratio (95% CI)	
*Acupuncture*				
Fatigue	0.54 (0.30-0.97)	.039	1.71 (1.18-2.49)	.005
Nausea	2.57 (0.97-6.81)	.057	1.86 (1.14-3.04)	.013
Neuropathy	6.59 (3.02-14.38)	<.001	2.25 (1.52-3.31)	<.001
Hot flashes	23.14 (3.08-173.91)	.002	2.08 (1.39-3.11)	<.001
Pain	4.15 (2.46-6.99)	<.001	2.17 (1.50-3.13)	<.001
Dry mouth	5.71 (1.92-16.99)	.002	2.07 (1.38-3.12)	<.001
Sleep	0.94 (0.52-1.68)	.825	1.50 (1.02-2.20)	.039
*Oncology massage*				
Fatigue	0.63 (0.30-1.34)	.230	1.17 (0.75-1.81)	.493
Neuropathy	1.18 (0.61-2.32)	.621	1.25 (0.8-1.95)	.322
Stress	2.20 (1.33-3.65)	.002	N/A	
Relaxation	2.74 (1.28-5.83)	.009	N/A	
Pain	3.04 (1.86-4.98)	<.001	2.14 (1.38-3.31)	.001
Sleep	0.49 (0.22-1.13)	.095	1.18 (0.75-1.87)	.472
Anxiety	N/A		1.60 (1.04-2.46)	.032
*Psychology referral*				
Anxiety	N/A		2.35 (1.41-3.90)	.001
Depression	N/A		2.08 (1.24-3.48)	.005
Fatigue	0.95 (0.43-2.11)	.899	1.80 (1.05-3.11)	.034
Pain	0.49 (0.22-1.05)	.068	1.00 (0.61-1.65)	.991
Stress	2.09 (1.18-3.70)	.012	N/A	
Sleep	1.83 (0.91-3.68)	.093	1.54 (0.88-2.70)	.131
Nausea	0.97 (0.28-3.39)	.960	1.96 (1.09-3.54)	.025
*Mind-body therapies*				
Anxiety	N/A		2.13 (1.47-3.09)	<.001
Depression	2.48 (0.93-6.65)	.070	1.54 (1.03-2.30)	.034
Stress/anxiety	2.57 (1.58-4.20)	<.001	N/A	
Fatigue	0.80 (0.45-1.42)	.447	0.87 (0.61-1.26)	.475
Pain	0.50 (0.31-0.80)	.004	0.65 (0.45-0.93)	.020
Sleep	0.90 (0.50-1.61)	.720	0.89 (0.61-1.30)	.553
Nausea	0.90 (0.37-2.22)	.821	1.13 (0.70-1.83)	.619

Abbreviations: ESAS, Edmonton Symptom Assessment System; IO, integrative oncology.

## Discussion

Our study highlights the real-world implementation of IO practices as a standard of care in a large comprehensive cancer center. An integrative approach, diet/nutrition, and pain were the top 3 reasons for IO referral by providers. In comparison, diet, pain, and an integrative approach were the top 3 concerns that patients wanted to discuss with IO providers as reported using the MYCaW. Referral for hot flashes and neuropathy or a symptom burden with an ESAS score of at least 4 for fatigue, nausea, neuropathy, hot flashes, pain, and dry mouth predicted acupuncture recommendation after IO consultation. Referral for pain and stress or an ESAS score of at least 4 for anxiety and pain predicted oncology massage recommendation. An interesting finding was the recommendation of mind-body therapies for patients referred for pain and an ESAS score of at least 4 for pain.^9,11,16-18^

Although the top 3 priorities of the providers and patients matched, we found an intriguing discrepancy between patient concerns and the reasons cited by providers for making referrals. This misalignment can be attributed to several factors that warrant further investigation. In a review of 21 studies, it was found that the prevalence of complementary and alternative medicine (CAM) use among cancer patients varied widely, ranging from 11% to 95%.^19^ Surprisingly, a significant portion of CAM users, ranging from 20% to 77%, did not disclose their CAM use to their healthcare providers (Davis et al). In a survey of 392 oncologists across US, it was found that, on average, oncologists discussed the use of herbal supplements (HS) with 41% of their patients, with only 26% of these discussions initiated by the oncologist. Notably, 2 out of 3 oncologists felt they lacked sufficient knowledge to answer patient questions about HS, and 59% had not received any education on the topic.^8^ The complex nature of patient-provider dynamics, individual perceptions of healthcare needs, varying perspectives on treatment options, and differing priorities could all contribute to this disconnect. Understanding the underlying reasons behind this incongruity is essential for optimizing patient-centered care and improving the alignment between patients’ concerns and providers referral decisions.

In our earlier analysis of IO consultations, patients’ top MYCaW concerns were an integrative approach (34%), herbs and supplements (34%), and diet/nutrition (21%).^20^ The present analysis suggested a shift in patient interests, with new top areas of interest including diet/nutrition, pain, and an integrative approach. Patients understand the significance of a healthy diet in relation to cancer and express a genuine interest in discovering optimal ways to modify their dietary habits to mitigate the risk of cancer recurrence or progression. They also recognize the potential impact of dietary choices on their overall well-being and actively seek guidance in adopting healthy dietary practices to support the journey toward improved health outcomes. This underscores the proactive role that patients play in their own health care as they strive to empower themselves with knowledge and take proactive steps toward reducing their cancer-related risks. National initiatives emphasizing the value of nonpharmacological approaches to pain management and updated pain management guidelines may have influenced both providers’ reasons for referral to and patients’ interest in IO interventions as adjuncts to pain management.^10^ Also of note is an overall higher symptom burden reported by referred patients at baseline compared to our prior study, in which we analyzed self-reported symptoms in our center from 2009 to 2013, including a pain score of 2.6 versus 3.5 in the present analysis of patients in 2017. A higher level of baseline pain at IO consultation in the present study than in our previous one may have contributed to our observation of why more patients had an interest in discussing pain as one of their primary concerns than in the previous study. Our findings demonstrate that implementing IO recommendations and guidelines in caring for patients with cancer is feasible.

Our study had some limitations. For example, it was a retrospective study at a large comprehensive cancer center. Therefore, our results may not be generalizable to IO practices across the globe, especially in community oncology settings. The patients were referred for IO consultation by oncology clinicians, and their settings may differ from those in which patients self-refer for IO clinical services/treatments. Also, we did not report on how availability or lack of insurance coverage may have influenced patient choice in pursuing treatments such as oncology massage or acupuncture or how a lack of health insurance coverage may limit access to IO services. Additional factors that can limit access to IO interventions include increased distance from our institution. We found that patients seen for IO consultation at our center lived a median distance of 98 miles away. Increased distance from our center may have led patients to pursue IO treatments such as massage or acupuncture locally rather than seeking a referral for treatment at our center. We did not account for patients who may have accessed these treatments in their local communities. In the future, we plan to explore treatment completion, in other words, how many patients referred to IO services completed their recommended courses of treatment (adherence) as part of their integrative care plans. New telehealth options for IO developed over the past several years may offer new opportunities to increase access to these interventions.^21^ In addition, health disparity research in IO settings is sparse, and our retrospective study did not address this issue. However, 23% of our study population was from underserved ethnic/racial groups.

## Conclusion

Our study highlights the real-world practice of IO and use of integrative therapies based on patients’ symptoms and reason for referral for IO consultations. An integrative approach, diet/nutrition, and pain were the top referral reasons and patients’ top concerns leading them to seek IO consultations. We found that acupuncture recommendation was predicted by a referral for hot flashes and neuropathy or patient self-reported significant fatigue, nausea, neuropathy, hot flashes, pain, or dry mouth. Oncology massage was predicted by a referral for pain and stress or patient-reported symptoms of anxiety and pain. Mind-body therapies were recommended for patients with pain. Future longitudinal research should focus on examining cancer patient’s symptom response to integrative therapies.
